# Effects of an Educational Glass Recycling Program against Environmental Pollution in Spain

**DOI:** 10.3390/ijerph16245108

**Published:** 2019-12-14

**Authors:** Miguel Ángel Aguilar-Jurado, Pedro Gil-Madrona, Juan Francisco Ortega-Dato, David Zamorano-García

**Affiliations:** 1 Faculty of Economic Sciences and Business Studies, University of Castilla-La Mancha, University Campus of Albacete, 02071 Albacete, Spain; migueldeestadistica@gmail.com (M.Á.A.-J.); juanfco.ortega@uclm.es (J.F.O.-D.); 2 Faculty of Education, University of Castilla-La Mancha, University Campus of Albacete, 02071 Albacete, Spain; david.zamorano@uclm.es

**Keywords:** pollution, glass recycling, environmental education

## Abstract

In this article, we analyzed the effects of an educational glass recycling program on primary schools and their students in Castilla-La Mancha (Spain). A sample of 89 schools, with 20,710 elementary students, was selected by simple random sampling. For the statistical analysis, descriptive techniques (frequencies and statistics), parametric (One Way ANOVA test), and non-parametric (Chi-Square test) inferential techniques were used, with a 5% significance level (*p* < 0.05). The program’s results showed that 153,576.3 kg of glass (with a value of 17,064.03 €) were recycled. Significant determinants of glass recycling were: School category (*p* = 0.043), previous environmental/recycling education (*p* = 0.046), geographic location of school (*p* = 0.030), gender (*p* = 0.007), and academic year (*p* < 0.05). With the program, students learned the importance of glass recycling, obtained a greater knowledge of and habits related to the same, acquired favorable attitudes towards the environment, and promoted glass recycling in their social circles. We conclude that environmental education about glass recycling has positive effects on glass recycling attitudes and behaviors in elementary school students and may be used as a measure to combat the problem of environmental pollution.

## 1. Introduction

Environmental pollution is one of the great challenges facing humanity today [1]. This global problem is defined as the presence of particular types and concentrations of physical, chemical, or biological agents in the environment that are harmful to the health, safety, and well-being of humans, plants, and animals [2]. The consequences of pollution are very worrying. According to the World Health Organization, 1.3 million people die annually due to high-level exposure to contaminating agents [3]. In urban areas, populations such as the poor, elderly, and children are especially prone to pollution-related diseases, including respiratory illnesses, heart disease, cancer, etc. [3,4,5,6,7,8,9,10,11,12]. Pollution generates other serious environmental problems such as climate change or global warming, which also have important health consequences [13,14]. Contamination is not only a threat to the environment and health of all living beings; it also affects global economic development [15]. In 2017, the estimated cost attributed to environmental pollution was greater than 4.6 billion dollars annually, the equivalent of 6.2% of the planet’s wealth [16].

In many societies, environmental pollution caused by waste is associated with the consumerism of a throw-away culture [17]. For example, in Spain, 75% of waste ends up in landfill sites, the equivalent of approximately 25 million tons annually. On average, each Spaniard produces 1.6 kg of waste per day, which represents 600 kg a year [18]. Of the huge amounts of residue generated at household, commercial, and industrial levels, glass is one of the most abundant disposable products, and its accumulation in the environment is of great concern.

Measures to reduce environmental pollution are multiple, for example: (1) Legal regulations that prohibit and sanction contaminating behaviors/activities, (2) fiscal measures (taxes, fees, etc.), (3) incentives to consume environmentally friendly goods and services (electric cars, public transport, and bicycles), (4) promotion of/subsidies for renewable energy sources, (5) investment in infrastructure and equipment (wastewater treatment plants, recycling, and waste management facilities, sewer systems, etc.), and (6) implementation of the 3 R’s (reduce, reuse, recycle). Recycling, in particular, reduces the accumulation of solid waste pollution by converting it into new products for reuse [19].

Glass recycling brings environmental, economic, and social benefits. Glass is 100% recoverable and can be endlessly recycled without losing its properties or functionality. With every 3000 standard-sized glass bottles recycled (75 cm [cl]/bottle), we save 1000 kg of waste accumulation [20]. From a health standpoint, glass recycling reduces air and water pollution, which translates into health benefits and savings in healthcare costs. In Spain, healthcare expenditures associated with pollution stood at 45 billion euros in 2013, 3.5% of the Usual abbreviation of Gross National Product (GNP). At the international level, this cost rises to 225 billion dollars (approximately 191 billion euros) [21]. Glass recycling reduces the amount of toxic particles and gases released into the air by about 20% [20] and at the same time, curbs greenhouse gas emissions or global warming. For every ton of recycled glass (cullet), 670 kg of carbon dioxide is saved from entering the atmosphere [22]. The quality of drinking water is also improved by about 50% due to glass recycling, reducing the risk of infection or chemical intoxication to the consumer [5]. Recycling glass also reduces land pollution and encourages a circular economy by lowering the consumption of natural resources necessary for glass production: Sand, limestone, sodium carbonate, etc. For example, 1 kg of cullet saves 1.2 kg of these materials from being mined, representing both an economic saving and reduced soil degradation [20,22]. Furthermore, compared to the raw materials necessary for glass manufacturing, cullet has a lower melting point, which results in an energy savings of 26.6% [20,23]. This translates into 136 L of petroleum saved for every ton of glass recycled [20]. Additionally, from a labor standpoint, recycling stimulates the economy by creating “green jobs” (jobs which reduce the environmental impact of industry and contribute to a sustainable economy). It is estimated that some 8000 jobs in Spain are directly or indirectly associated with glass recycling [24].

Given the problems that pollution causes and the advantages of recycling, society should adopt this practice as normal behavior. Conscious of this, the European Union has imposed, as part of its environmental strategy, that member states recycle 55% of all waste generated by the year 2025 [25]. Countries like Spain, with a recycling rate of 29.7%, are still far from achieving this goal and must continue to work towards it [26].

In order to encourage recycling, we need to understand which factors determine the intention to recycle. These factors can be grouped into: (1) Demographic factors: Age, gender, education level, income level, marital status, ethnic group, geographic location, etc. [27,28,29,30,31,32], (2) psychographic factors: Knowledge, motivation and environmental attitudes and concern, etc. [30,31,33,34,35], and (3) circumstantial factors: Convenience, information, variety of recycling options available, access to recycling opportunities and containers, imitation of other individuals, social norms, economic obligations, etc. [31,33,35,36]. Regarding glass recycling, the following factors have also been found to influence the intention to recycle: Moral values, ecological consciousness, and past behavior [37].

Environmental education, which includes recycling programs, plays an important role in fostering and transmitting pro-environmental knowledge, consciousness, attitudes, and behavior [38]; it should be understood as a lifelong, continuous educational process whose foundations are laid down in childhood. It has been shown that it is more effective to instill environmental consciousness in preadolescent children who have not yet developed well-established environmental habits [39]. Therefore, environmental education programs that encourage the development of positive environmental attitudes and behaviors should be incorporated into the primary school curriculum [40].

Currently, there are many environmental education programs with diverse contents. Over the years, many international organizations concerned with protecting the environment have instigated educational programs, such as: *GAP -Global Action Plan-* [41], *Western Mediterranean Sea Project* [42], *Clean Up the World* [43], *Eco-School* [44], *GLOBE -Global Observations for the Benefit of the Environment-* [45], *Ocean Initiatives* [46], *Conectando Mundos* [47], etc. In Spain, according to figures from the Ministry for Ecological Transition (MITECO) [48], there are a total of 132 official environmental education programs, such as: *Ecoauditoría*, *Escuelas amigas de los bosques*, *Aldea*, *Actúa con energía*, *Valles del oso*, *PINEA*, *¡Explora tu río!*, *Conocer nuestro medio*, *Aula del fuego*, *Escoles verdes*, *Climática*, *Aulas en las montañas*, *Agenda 21 Escolar*, etc. These programs offer diverse teaching approaches and content matter; some are more general and others more specific (climate change, protection of rivers, mountains, forests, beaches, animal species, etc.). According to scientific evidence, we know that environmental attitudes of a general nature are predictors of specific environmental attitudes amongst students. However, the general environmental outlook does not appear to be significantly correlated to specific environmental behaviors such as recycling [36]; therefore, specialized programs rather than general environmental education may be required to achieve specific behaviors.

In Spain, recycling education programs are not abundant, much less those dealing exclusively with glass. According to MITECO [48], the following official recycling programs can be found: (1) *Profesor Reciclus*, a Gredos San Diego Foundation project aimed at teaching first-grade elementary students the principles of Reduce, Reuse, Recycle, (2) *Recapacicla*, a Government of Andalusia program about waste and recycling, (3) *La Aventura de tu Basura*, a motivational program dealing with source separation of household waste and recycling, taught in schools in Córdoba, and (4) *Red de Escuelas por el Reciclaje*, initiated by the Consortium for Solid Waste Management in Asturias, seeking to foment the 3 Rs (Reduce, Reuse, Recycle) in the educational community. In addition to these official programs, there are others designed by private environmental organizations, such as: (1) *Educa en Eco*, dedicated to recycling paper, cardboard, plastic, cans, and cartons in schools [49], and (2) *La Liga Peque Recicladores*, focused on glass recycling in the school environment [50].

There is little scientific research on the topic of recycling education programs. There are, however, some relevant studies in this area, such as that of Pellegrini and Reyes [51], analyzing a paper and cardboard recycling program geared toward the student population of the Simón Bolívar University in Venezuela during the 2004–2007 period. Besides raising awareness and fomenting pro-environmental attitudes amongst university students, considerable quantities of material were recycled due to the program: Specifically, 3110 kg of paper and 617 kg of cardboard. We discovered another interesting study by Romero, Salas, and Jiménez [52], who evaluated the MADI (Management of Institutional Waste) program at the Technological Institute of Costa Rica during the years 2000–2007. The results were satisfactory in terms of the 3 R’s (reduce, reuse, recycle) and responsible disposal of ordinary solid waste (plastic, glass, paper, and aluminum). The program also raised environmental awareness in the institute’s student body, administration, and teaching community. We should also mention the work of Ponte [53], regarding the paper recycling project at the Pedagogical Institute of Caracas (Venezuela), which resulted in the recovery of 31,181 kg of paper for recycling during the years 2000–2006. This activity allowed the institute to become financially self-sufficient and the model of a sustainable university. In Australia, Armstrong, Sharpley, and Malcolm [54], Armstrong and Grant [55], and Cutter-Mackenzie [56] conducted a scientific review of the program Waste Wise Schools. This is an environmental education program based on the 3 R’s for primary and secondary schools in Western Australia. The main results of this program, since its conception in 1997, have been the reduction of miscellaneous waste (plastic, cardboard, paper, glass, organic waste, etc.) and the development of positive environmental attitudes in students and the school community through practical learning experiences. Finally, Buil, Roger-Loppacher and Prieto-Sandoval [57] studied the effects of an educational program (theoretical and practical) about aluminum packaging recycling. The program was organized by ARPAL (Association for the Recycling of Aluminum in Spain), in 2015, for students between 8 and 12 years old, from Avila and Cadiz (Spain). The study showed that after participating in the program, students had increased knowledge and awareness of aluminum recycling and greater intention to recycle aluminum.

In summary, given the importance of the topic at hand and the scarcity of glass recycling programs and scientific research pertaining to them, the present work analyzed the glass recycling education program, *La Liga Peque Recicladores*. We intended to contribute to the scientific literature by investigating the effects of a glass recycling education program and by demonstrating some of the factors determining this type of recycling. The specific objectives of this study were: (1) To quantify the amount of glass collected for recycling and calculate its economic value, (2) to evaluate if the type of school, previous environmental/recycling education, and geographic location were influential factors in glass recycling; (3) to determine, using a psychometric scale, if students improved in terms of importance given to glass recycling, knowledge of the topic, recycling habits, respect for the environment, and promotion of glass recycling in their social circles, and (4) to examine whether there were significant differences according to gender and academic year in the above-mentioned recycling attitudes as measured by the psychometric scale.

## 2. Materials and Methods

### 2.1. Description of the Program and Procedures

*La Liga Peque Recicladores* (The League of Little Recyclers) refers to an environmental education program about recycling, organized by Ecovidrio [50]. It consisted of a glass collection competition and other complementary activities with the goal of informing and sensitizing primary school children to the importance and benefits of glass recycling. Furthermore, the program’s objective was to foment recycling behaviors in this population, their families, and the educational community.

The program was carried out in public, private, and government-subsidized private schools (semi-private) between November 2017 and January 2018, in the Spanish regions of Castilla-La Mancha, Miranda del Ebro, Ponferrada, and Salamanca. The current study focused its analysis on the Community of Castilla-La Mancha, including the following municipalities: Almansa, Ciudad Real, Cuenca, Guadalajara, Hellín, Puertollano, Talavera de la Reina, and Toledo.

*La Liga Peque Recicladores* program was divided into 5 phases: (1) Announcement of the program, when elementary schools from the above-mentioned municipalities of Castilla-La Mancha were contacted via e-mail and telephone to inform them of the objectives and characteristics of the program, (2) Incorporation of participating schools through electronic registration on the program’s webpage, (3) Placement of vinyl stickers with the program’s logo on the glass recycling bins closest to participating schools, (4) Advertisement of the program through publicity (posters, flyers, webpage, school events) and educational activities such as theatre and school workshops, (5) Monthly rankings and interim prizes, where each month, the number of kilograms of glass collected per student in the recycling containers was tabulated; the interim prizes (a visit to a children’s play center) were awarded to the school collecting the most glass/student in that particular month, and (6) Determination of final rankings and distribution of prizes (monetary awards for scholastic material, sports equipment or computers) when schools were classified in terms of the accumulated number of kilograms of glass/student collected for recycling throughout the 3 months of the program. Monetary prizes in the amounts of 3000, 2000, or 1000 euros, according to 1^st^, 2^nd^, or 3^rd^ placement, were awarded to the 3 winning schools.

After completing the program *La Liga Peque Recicladores*, students’ educational experience was evaluated by means of a questionnaire distributed amongst the participating schools regarding knowledge, attitudes, the importance of, and habits pertaining to glass recycling.

Finally, once the results of the program were analyzed and considered satisfactory, we agreed to publish the data.

### 2.2. Study Design

A cross-sectional study design was employed, with both descriptive and inferential statistics.

### 2.3. Sample

Of the 153 primary education schools in Castilla-La Mancha, a total of 89 were sampled by simple random sampling (*p* = *q* = 50%; margin of error = 6.7%; confidence level = 95%). Table 1 shows the selected sample according to the school category and municipality of Castilla-La Mancha. The sample was comprised of 68.54% public, 30.34% semi-private, and 1.12% private schools, distributed throughout 8 municipalities of Castilla-La Mancha: Almansa (7%), Ciudad Real (15%), Cuenca (17%), Guadalajara (11%), Hellín (9%), Puertollano (10%), Talavera de la Reina (15%) and Toledo (17%). Of these schools, 14.61% had provided previous environmental/recycling education to students, and all of them were concentrated in the municipality of Talavera de la Reina (100%). Table 2 shows the demographic characteristics (surface area, population density, and per capita income) of the municipalities of Castilla-La Mancha sampled for this study [58].

The sampling unit was defined as the education centers; however, the students at the selected schools were also studied because of their relevance to the current research. The schools in our sample were comprised of a population of 20,710 students, of which 58.17% participated in the program; 48.8% of these students were girls, and 51% were boys. The students we studied were in elementary school, grades 1 to 6 (between the ages of 6 and 12).

### 2.4. Variables

Table 3 shows the variables defined in the study, grouping them together under the following headings: (1) Physical quantification of the glass collected for recycling (in kilograms [Kg]), (2) economic value of the said glass (in euros [€], according to market price [75 cl glass bottle = 0.05 €/1 Kg glass = 0.11 €]) [59], (3) psychometrics of glass recycling measured by a Likert scale, (4) classification of schools by category (public, private and semi-private), previous environmental/recycling education (Yes/No), geographic location (municipality), and (5) classification of students by gender (female and male) and academic year (elementary school, grades 1 to 6). The variables of groups 1–3 were the dependent variables in this study, and those of groups 4 and 5, the independent variables.

### 2.5. Instruments

The psychometric variables were obtained by means of an ad hoc questionnaire, based on the instrument Validity and reliability of the scale of attitudes towards the recycling and responsible use of paper in students of the *Universidad Nacional Mayor de San Marcos* (*UNMSM*) [60]; terminology and content were adapted to the context of glass recycling and to the comprehension level of elementary school students.

The validity of the scale was satisfactorily demonstrated by the construct method, using internal consistency and total test (Table 4). Reliability was tested by Cronbach’s alpha formula (Table 5).

After completing the *La Liga Peque Recicladores* program during the 2017–2018 academic year, students were questioned, using the psychometric instrument, about the importance of glass recycling as well as about knowledge, attitudes, and habits pertaining to glass recycling. Results were compared to the 2016–2017 school year, in which the program was not carried out. A 3-point, 20-item Likert scale was employed (1 = more than last school year, 2 = equal to last school year, 3 = less than last school year), which was organized into 5 sub-scales about glass recycling (4 items/sub-scale): (1) Importance, (2) Knowledge, (3) Habits, (4) Respect for the Environment, and (5) Promotion/Interaction. The instrument also contained questions classifying the students by gender and academic year.

### 2.6. Statistical Analysis

For the statistical analysis of the data, we used descriptive techniques such as frequencies (absolutes and percentages [%]), arithmetic means (M), and standard deviations (SD). Parametric inferential methods such as the one-way ANOVA were also utilized (F-statistic, degrees of freedom [df1, df2] and *p*-value [*p*]), with effect size measured by Eta-squared ($\eta^{2}$). Additionally, the data analysis included the non-parametric chi-square test (chi-square statistic [$\chi^{2}$], degrees of freedom [df], and *p*-value [*p*]), with effect size measured by the contingency coefficient (C). A significance level of 5% was employed in all statistical tests (*p* < 0.05).

A global statistical analysis was undertaken for the school sample, with the variables school category, previous environmental/recycling education, and geographic location; likewise, a global analysis was employed for the student sample, with the variables gender and academic year.

The statistical programs SPSS (Statistical Software Package for the Social Sciences) and R were used to process the data.

### 2.7. Ethics Statement

The present study was developed respecting the requirements of the Ethics Committee of the University of Castilla-La Mancha (Spain).

## 3. Results

As a result of *La Liga Peque Recicladores,* a total of 153,576.3 kg of glass were recycled in the locations studied in Castilla-La Mancha, with an average of 2344.15 kg of glass/school (SD = 3242.4); the glass recycling rate per student was 7.42 kg.

**Figure 1:**
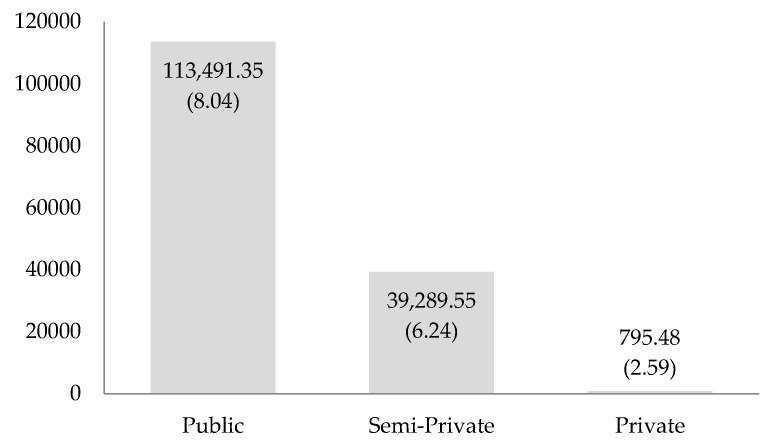
Total quantity and rate per student of recycled glass according to the school category.

Significant differences (95% confidence level) were observed in terms of rates of glass recycled per student according to school category (F = 3.245; df1 = 2; df2 = 86; *p* = 0.043; $\eta^{2}$ = 0.191). Figure 1 compares the total quantity and rate per student (in parentheses) of recycled glass according to the school category. Public schools recycled the most, with a total quantity of 113,491.35 kg (74%), an average of 2527.46 kg of glass/school (SD = 3810.45), and a rate of 8.04 kg of glass/student.

**Figure 2:**
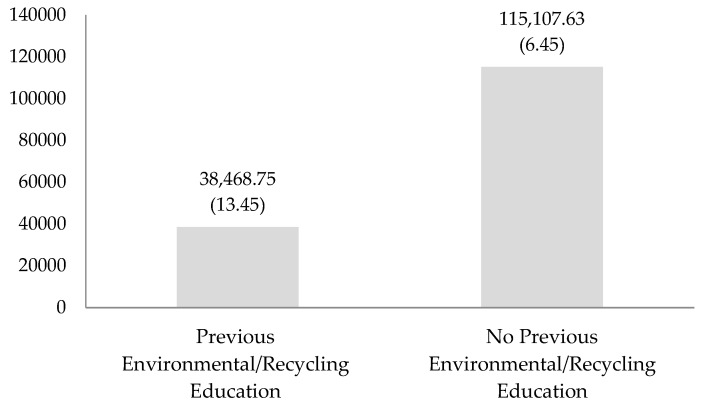
Total quantity and rate per student of recycled glass according to previous environmental/recycling education.

Significant differences (95% confidence level) in glass recycling rates per student were also detected as a function of previous environmental/recycling education in schools (F = 4.102; df1 = 1; df2 = 87; *p* = 0.046; $\eta^{2}$ = 0.154). Figure 2 compares the total quantity and rate per student (in parentheses) of recycled glass according to previous environmental/recycling education. Schools with previous training recycled the least in terms of total amount of glass collected, which was 38,468.75 kg (25.05%); however, they obtained a higher average collection per school, with 4019.91 kg of glass (SD = 4960.06) and a higher recycling rate per student (13.45 kg of glass/student).

**Figure 3:**
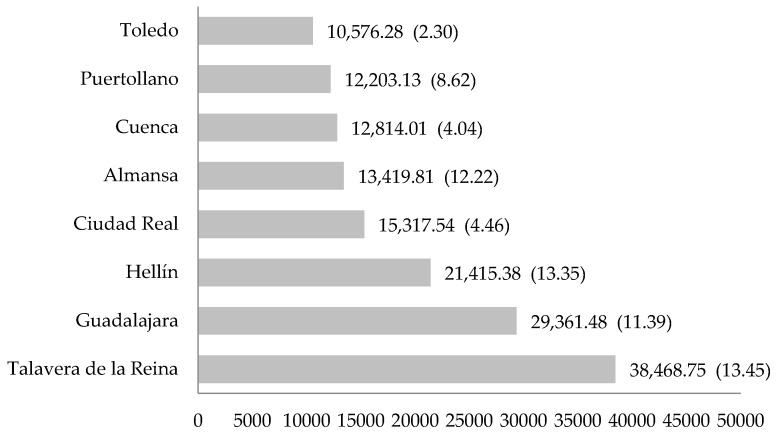
Total quantity and rate per student of recycled glass according to geographic location.

According to geographic location, there were also significant differences (95% confidence level) in glass recycling rates per student (F = 2.362; df1 = 7; df2 = 81; *p* = 0.030; $\eta^{2}$ = 0.169). Figure 3 compares the total quantity and rate per student (in parentheses) of recycled glass according to geographic location. Schools from the municipality of Talavera de la Reina recycled the most, with a total quantity of 38,468.75 kg of glass (25.05%), an average of 4019.91 kg of glass/school (SD = 4960.06), and a rate of 13.45 kg of glass/student.

From an economic standpoint, the total quantity of glass recycled was valued at 17,064.03 €, an average of 260.46 € (SD = 360.31) per school, and a contribution of 0.82 € per student. The public schools stood out, with a rate of 0.89 € per student and a total amount of glass worth 12,610.14 €, with an average of 280.83 €/school (SD = 423.38). Schools with previous environmental/recycling education also showed high levels of recycling, with a rate of 1.49 € per student and an average of 446.66 €/school (SD = 551.12). According to geographic location, the schools in Talavera de la Reina recycled the most, with a rate of 1.49 € per student and a total amount of glass collected worth 4274.30 €, with an average of 446.66 €/school (SD = 551.12).

With respect to the psychometrics of glass recycling, overall results (Table 6) showed that 65.2% of participating students gave greater importance to the task of glass recycling after completing the *La Liga Peque Recicladores* program. Furthermore, 69.3% obtained more knowledge about the topic, 55.3% claimed to have acquired glass recycling habits, 68% professed a more respectful attitude towards the environment, and 64.8% indicated higher levels of promotion and social interaction with respect to glass recycling in their circle of family and friends.

With respect to gender and the psychometric scale (Table 7), significant differences were observed (95% confidence level) only for the variable *Habits* ($\chi^{2}$ = 10.058; df = 2; *p* = 0.007; C = 0.199), where girls obtained better results than boys.

According to the academic year (Table 8), there were significant differences (95% confidence level) in the following subscales of the psychometric instrument: (1) *Importance* ($\chi^{2}$ = 22.387; df = 10; *p* = 0.013; C = 0.290), (2) *Knowledge* ($\chi^{2}$ = 21.616; df = 10; *p* = 0.017; C = 0.285), (3) *Habits* ($\chi^{2}$ = 38.088; df = 10; *p* < 0.05; C = 0.367), (4) *Respect for the Environment* ($\chi^{2}$ = 23.358; df = 10; *p* = 0.010; C = 0.296), and (5) *Promotion/Interaction* ($\chi^{2}$ = 36.492; df = 10; *p* < 0.05; C = 0.361). Specifically, first graders performed best in terms of *Importance*, fourth graders in terms of *Knowledge* and *Respect for the Environment*, second graders stood out in *Habits*, and third graders in *Promotion/Interaction*. The highest scores on our scale were found in the earlier stages of elementary school (grades 1–4), while the worst performance was observed in grades 5 and 6, especially in the last academic year.

## 4. Discussion

The environmental education program, *La Liga Peque Recicladores* has turned out to be an innovative and unique project of its kind in Spain. This study demonstrates satisfactory results in glass bottle collection for recycling in elementary school students from Castilla-La Mancha, from an educational, environmental, economic, and social perspective.

The glass recycling activities of this program allowed elementary school students to contribute to society by respecting and protecting the environment as we face a global pollution problem. Students learned to appreciate the importance of glass recycling, obtained more knowledge on the topic, and acquired environmentally proactive attitudes and respectful behaviors/habits. These results coincide, to a large extent, with evidence from other recycling education programs that increased recycling behavior amongst students and stimulated positive environmental awareness, knowledge, and attitudes [39,51,52,53,54,55,56,57,61]. Furthermore, the students in our study promoted glass recycling within their networks of family and friends, which helps to widen the scope of these education programs [54,62].

From an economic point of view, if the glass collected from the *La Liga Peque Recicladores* program were sold on the market, the income generated could lead to self-financing of this educational activity; similar paper recycling education programs have already adopted this method of self-funding [29]. Furthermore, this glass recycling program is not only capable of generating income, but it could also lead to economic savings in the high healthcare and environmental costs resulting from pollution [63,64,65,66,67,68,69,70,71,72,73]. Therefore, governments should pay attention to the link between health and the environment when formulating public policies; likewise, it is incumbent upon the private industry to develop socially responsible behaviors.

Apart from analyzing the program´s results, another objective of this investigation was to study the determining factors of glass recycling behavior, given the scarcity of scientific evidence about glass recycling. In this regard, our study reveals that the school category, geographic location, previous environmental/recycling education, gender, and academic year influence glass recycling behavior.

In terms of school category, the results of the program show that public school students recycled more than students from private or semi-private schools, reinforcing the theory that the type of school and teaching program influence the recycling of materials such as paper [39] or, in our case, glass. Possible reasons for this difference in behavior are the priority that private and semi-private centers give to scholastic achievement, which discourages participation in extra-curricular programs, and teachers´ lack of time and commitment [74,75,76,77]. Therefore, the importance of a broad education needs to be transmitted to these centers, with the incorporation of complementary subjects such as environmental education and recycling.

Geographic location is another factor influencing glass recycling behavior. The program shows that municipalities with a smaller surface area, greater population density, and a lower per capita income, such as Talavera de la Reina, recycled more than other communities analyzed in the region of Castilla-La Mancha. However, upon closer inspection, the municipality with the second-highest level of recycling was Hellín, characterized by a larger surface area and a lower population density, in contrast to Talavera de la Reina. Nonetheless, both municipalities coincide in lower levels of income per capita. Therefore, of the above-mentioned demographic characteristics, the economic factor appears to be the most influential in determining recycling behavior, confirming the results of previous studies [29].

Furthermore, according to the program, previous environmental/recycling education also influences glass recycling behavior. Schools in municipalities that were hosts to past programs, such as Talavera de la Reina with *Conocer nuestro medio* [48], an environmental education program with considerable recycling content, recycled more glass compared to the rest. This supports the theory that prior specialized education is required in order to develop specific behaviors such as recycling habits [36], and that past conduct is a relevant factor influencing intention to recycle glass [37]. Talavera de la Reina also has lower levels of income per capita; therefore, it appears that reduced monetary resources in some municipalities lead them to implement lower-cost, but effective, recycling policies that included education programs in schools. The potential long-term repercussions of these programs are important since recycling habits learned in childhood may influence adult recycling behavior.

In terms of gender as a factor related to glass recycling, the study demonstrates that female students acquired better glass recycling habits than males. According to research by the authors Brough et al. [78], pro-environmental behaviors are more strongly associated with women than with men; in fact, males may even reject these behaviors in order to preserve an image of masculinity. This can be explained by the influence of social norms, which appear to have a greater impact than morals or ethics upon environmental behaviors such as recycling [29]. We recommend that future programs introduce elements of equality and destigmatization in order to avoid gender-biased behavior.

With respect to the academic year, students in grade 2 (7–8 years old) presented the best glass recycling habits, which diminished with each subsequent stage in primary school education, especially in grades 5 (10–11 years old) and 6 (11–12 years old). This reveals the need to reinforce recycling habits as children grow [79], and to make environmental education and recycling training a continuous process throughout the years of formal education [80].

Finally, since our research presents some limitations, the results should be interpreted with caution. Firstly, the study area focuses on a small part of Spain. It would be advisable to develop similar, new studies covering more territory and in other countries, in order to compare results and obtain more significant effects. Another problem area of this study concerns data collection. The glass recycling program only quantifies the amount of glass recycled by the school and geographic area. Demographic variables such as gender and academic year are excluded from these statistics and are only correlated to the psychometrics of the glass recycling questionnaire. Therefore, future studies should take such demographic variables into account when measuring the amount of glass recycled. A further limitation of this study is the length of the program, which is only 3 months. In order to obtain more reliable evidence, recycling education programs should be of longer duration.

## 5. Conclusions

Environmental education about glass recycling has positive effects on glass recycling attitudes and behaviors in elementary school students and may be used as a long term measure to combat the problem of environmental pollution, especially if its geographical reach is widened. To improve these effects, factors such as school category, previous environmental/recycling education, geographic location, gender, and the academic year must be considered.

## Figures and Tables

**Table 1:** Participating schools according to category and municipality in Castilla-La Mancha.

Municipality	School Category			
	Public	Semi-Private	Private	Total
Almansa	4	2	0	6
Ciudad Real	10	3	0	13
Cuenca	14	1	0	15
Guadalajara	8	2	0	10
Hellín	5	3	0	8
Puertollano	7	2	0	9
Talavera de la Reina	4	9	0	13
Toledo	9	5	1	15
Total	61	27	1	89

**Table 2:** Demographic characteristics of municipalities sampled in Castilla-La Mancha.

Municipality	Surface Area (km^2^)	Population Density (People/km^2^)	Per Capita Income (€)
Almansa	531.91	46.62	20,249
Ciudad Real	285.15	259.70	27,271
Cuenca	911.06	60.48	24,570
Guadalajara	235.48	355.16	26,628
Hellín	781.19	38.94	17,313
Puertollano	226.74	216.84	24,991
Talavera de la Reina	185.83	452.67	20,368
Toledo	231.76	360.11	26,296

**Table 3:** Variables.

Quantity of Glass for Recycling	Economic Value of Glass for Recycling	Psychometrics of Glass Recycling	Classification
Total Kg Glass	Total Euros Glass	Importance	School Category
Kg Glass/School	Euros Glass/School	Knowledge	Previous Education
Kg Glass/Student	Euros Glass/Student	Habits	Geographic Location
		Respect for the Environment	Gender
		Promotion/Interaction	Academic Year

**Table 4:** The validity of the scale demonstrated by internal consistency and total test.

Sub-Scales	Importance	Knowledge	Habits	Respect for the Environment	Promotion/Interaction	Total
Importance	1					
Knowledge	0.198 **	1				
Habits	0.323 **	0.359 **	1			
Respect for the Environment	0.421 **	0.389 **	0.527 **	1		
Promotion/Interaction	0.380 **	0.287 **	0.334 **	0.415 **	1	
Total	0.656 **	0.622 **	0.734 **	0.781 **	0.707 **	1

(**) *p* < 0.01.

**Table 5:** Reliability of the scale tested by Cronbach’s alpha.

Sub-Scales	Cronbach’s Alpha
Importance	0.712
Knowledge	0.724
Habits	0.821
Respect for the Environment	0.741
Promotion/Interaction	0.703
Total Scale	0.740

**Table 6:** Psychometrics of glass recycling for the total number of participating students (percentages).

	Comparison with Previous School Year			
	More	Equal	Less	Total
Importance	65.2	34	0.8	100
Knowledge	69.3	29.9	0.8	100
Habits	55.3	41.8	2.9	100
Respect for the Environment	68	30.4	1.6	100
Promotion/Interaction	64.8	30.7	4.5	100

**Table 7:** Psychometrics of glass recycling for participating students according to gender (percentages).

Gender	Comparison with Previous School Year			
Females	More	Equal	Less	Total
Importance	63.9	36.1	0.0	100
Knowledge	71.4	27.7	0.9	100
Habits	65.5	32.8	1.7	100
Respect for the Environment	74.8	23.5	1.7	100
Promotion/Interaction	66.4	31.1	2.5	100
Males	More	Equal	Less	Total
Importance	66.4	32.0	1.6	100
Knowledge	67.2	32.0	0.8	100
Habits	45.6	50.4	4.0	100
Respect for the Environment	61.6	36.8	1.6	100
Promotion/Interaction	63.2	30.4	6.4	100

**Table 8:** Psychometrics of glass recycling for participating students, according to the academic year (percentages).

Academic Year	Comparison with Previous School Year			
Grade 1	More	Equal	Less	Total
Importance	76.5	23.5	0.0	100
Knowledge	79.4	20.6	0.0	100
Habits	67.7	29.4	2.9	100
Respect for the Environment	76.5	20.6	2.9	100
Promotion/Interaction	76.5	20.6	2.9	100
Grade 2	More	Equal	Less	Total
Importance	56.8	43.2	0.0	100
Knowledge	75.7	24.3	0.0	100
Habits	73.0	21.6	5.4	100
Respect for the Environment	73.0	27.0	0.0	100
Promotion/Interaction	67.6	29.7	2.7	100
Grade 3	More	Equal	Less	Total
Importance	73.8	26.2	0.0	100
Knowledge	61.9	35.7	2.4	100
Habits	69.0	28.6	2.4	100
Respect for the Environment	69.0	31.0	0.0	100
Promotion/Interaction	78.6	19.0	2.4	100
Grade 4	More	Equal	Less	Total
Importance	72.7	27.3	0.0	100
Knowledge	84.1	15.9	0.0	100
Habits	65.9	31.8	2.3	100
Respect for the Environment	81.8	13.6	4.6	100
Promotion/Interaction	77.3	20.5	2.2	100
Grade 5	More	Equal	Less	Total
Importance	66.7	28.9	4.4	100
Knowledge	71.1	28.9	0.0	100
Habits	26.7	68.9	4.4	100
Respect for the Environment	64.4	33.3	2.3	100
Promotion/Interaction	55.6	31.1	13.3	100
Grade 6	More	Equal	Less	Total
Importance	45.2	54.8	0.0	100
Knowledge	45.2	52.4	2.4	100
Habits	35.7	64.3	0.0	100
Respect for the Environment	45.2	54.8	0.0	100
Promotion/Interaction	35.7	61.9	2.4	100
